# Complete genome sequence of a Bangladesh-developed live-attenuated goatpox (GTPV) vaccine strain

**DOI:** 10.1128/mra.00192-26

**Published:** 2026-05-18

**Authors:** Anowar Hossen, Mohammed A. Samad, Md. Rezaul Karim, A.S.M. Ashab Uddin, Mst Nazia Akter, Khairun Nahar Shithi, Paul W. Selleck, Dieter M. Bulach, Timothy R. Bowden

**Affiliations:** 1 Vaccine and Biologics Laboratory, Transboundary Animal Disease Research Center, Bangladesh Livestock Research Institute, Savar, Dhaka, Bangladesh; 2 Animal Health Research Division, Bangladesh Livestock Research Institute, Savar, Dhaka, Bangladesh; 3 CSIRO Australian Centre for Disease Preparedness 64808 , Geelong, Victoria, Australia; 4 The Asia-Pacific Centre for Animal Health, Melbourne Veterinary School, The University of Melbourne 2281, Parkville, Australia; Portland State University, Portland, Oregon, USA

**Keywords:** complete genome sequence, goatpox virus, goat, Bangladesh

## Abstract

Goatpox is an economically important transboundary disease affecting goats in Bangladesh, which is primarily managed through a live-attenuated goatpox vaccine. Here, we report the complete genome sequence of a Bangladesh-derived goatpox virus (GPV) vaccine strain generated through serial cell culture passages.

## ANNOUNCEMENT

Goatpox virus (GPV), a member of the genus *Capripoxvirus* (family *Poxviridae*), often causes fatal disease in goats (1). In Bangladesh, vaccination is widely used to control goatpox.

A live-attenuated goatpox vaccine was developed from a locally circulating GPV strain (GTP-43, accession no. PX499192) isolated from goats in Meherpur, Bangladesh in 2021. The virus was attenuated through serial passaging: initially in primary lamb testis cells (five passages), followed by 35 passages in Vero cells (American Type Culture Collection [ATCC] CCL-81, USA) and 25 passages in MDBK cells (ATCC CCL-22, USA). Cultures were performed under conditions using a multiplicity of infection (MOI) of 0.01 at 37°C with 5% CO_2_. Cells were maintained in minimum essential medium (cat. no. 11430-030, Gibco, USA) supplemented with 1% fetal bovine serum (cat. no. SV30160.03, HyClone, Austria). Viral harvest was performed at approximately 80% cytopathic effect using repeated freeze–thaw cycles. Attenuation of multiple passaged virus was evaluated by back-passages in young goats, with no observable adverse clinical effects, and induction of a detectable antibody response, as described previously (2–4). Here, we present the complete genome sequence of the Bangladesh goatpox vaccine strain (accession no. PX734138.1). The complete genome sequence was determined using next-generation sequencing (NGS) on the Illumina platform by aligning it with the reference complete genome sequence of goatpox virus (NC_004003).

Viral DNA used for sequencing was extracted from culture supernatant of infected MDBK cells, which were infected at a MOI of 0.01 and incubated for 2–3 days until approximately 80% cytopathic effect. The supernatant was collected by harvesting and clarification via low-speed centrifugation prior to DNA extraction. Viral DNA was isolated from the culture supernatant of Bangladesh goatpox vaccine strain using the PureLink Viral DNA Mini Kit (Invitrogen, USA) following the manufacturer’s protocol. DNA concentration was measured with the Qubit dsDNA Quantification Assay yielding 15.1 ng/µL, and 350 ng was used for library construction. Libraries were prepared with the Illumina DNA Prep Kit (Illumina, USA) and sequenced on an Illumina NextSeq 2000 platform, generating ~400 bp inserts. Paired-end sequencing (2 × 150 bp) produced 14.57 million reads. Read quality was evaluated using FastQC v0.11.3 (5). Adapter sequences and low-quality bases were trimmed using Trimmomatic v0.39.2 (6), applying the following parameters: minimum Phred quality score of >30, minimum read length of >35 bp, and 5′ clipping of 15 bases for both forward and reverse reads.

Host reads were removed using Kraken2 with the standards RefSeq database (7), and 284,498 GPV reads were assembled with SPAdes v3.15.5 employing k-mer sizes of 53, 65, 75, and 83. The genome was assembled as a single contig of 150,207 bp with a median depth coverage of 284× and 150 predicted open reading frames with an average guanine-cytosine (GC) content of 41% (8). The terminal hairpin loop structures of this genome were not resolved in this assembly. Genome annotation was performed using Prokka, which incorporates Prodigal for gene prediction, and results were manually curated by comparison with goatpox virus reference genome (accession NC_004003) (9). Comparative variant analysis was performed using Snippy v4.4.5. All software tools were run with default parameters unless otherwise specified. The Bangladesh goatpox vaccine strain and its parental strain, Bangladesh GTPV-43, exhibited 99.98% nucleotide sequence identity and differed by five nucleotide changes, comprising one insertion, one deletion, and three single nucleotide polymorphisms. Four of these variations occurred within coding regions (Table 1). These mutations affect specific amino acid positions, possibly leading to changes in protein structure and function.

**TABLE 1:** Genome-wide variants identified by Snippy between the goatpox vaccine strain PX734138 and parent Goatpox virus isolate (PX499192) in Bangladesh

POS^*a*^	Type^*b*^	REF^*c*^	ALT^*c*^	NT_POS^*d*^	AA_POS^*d*^	Effect^*e*^	LOCUS_TAG	Product
15,691	ins	C	CTA					
53,910	del	CG	C	267/279	89/92	frameshift_variant c.267delG p.Ser90fs	GTPV_BLRI_S043_2021_Bangladesh_00057	Crescent formation protein
96,604	snp	C	A	634/2715	212/904	missense_variant c.634G>T p.Val212Phe	GTPV_BLRI_S043_2021_Bangladesh_00097	Hypothetical protein
99,432	snp	G	T	152/162	51/53	stop_gained c.152C>A p.Ser51*	GTPV_BLRI_S043_2021_Bangladesh_00102	IMV membrane receptor-like protein
101,366	snp	C	T	212/585	71/194	missense_variant c.212G>A p.Gly71Glu	GTPV_BLRI_S043_2021_Bangladesh_00105	IMV membrane protein

^*a*^ POS, position of mutation in parent isolate genome.

^*b*^ Type, mutation type; ins—insertion, del—deletion, snp—single-nucleotide polymorphism.

^*c*^ REF, sequence in the Bangladesh goatpox GTPV-43 isolate; ALT: sequence in the Bangladesh goatpox vaccine isolate.

^*d*^ NT_POS and AA_POS represent nucleotide and amino acid positions relative to coding sequences, shown as parent/vaccine coordinates. IMV: intracellular mature virion.

^*e*^ Change in encoded protein; standard three-letter amino acid code is used.

## Data Availability

The complete genome sequence of the Bangladesh-developed live-attenuated Goatpox (GTPV) vaccine strain has been submitted to GenBank under accession number PX734138.1. The raw sequence reads have been submitted to the SRA database under accession number SRR37317723.
